# Multiplex Antibody Measurement for Post-treatment Follow-up of Staphylococcal Prosthetic Joint Infection: A Diagnostic Pilot Study

**DOI:** 10.7150/jbji.36015

**Published:** 2019-09-26

**Authors:** Thomas BAUER, Simon MARMOR, Idir GHOUT, Elsa SALOMON, Faten EL SAYED, Beate HEYM, Martin ROTTMAN, Jean-Louis GAILLARD, Anne-Laure ROUX

**Affiliations:** 1 Service de Chirurgie Orthopédique et Traumatologie, Hôpital Ambroise Paré (Assistance Publique - Hôpitaux de Paris, AP-HP), Boulogne-Billancourt, France.; 2 Centre de référence des infections ostéo-articulaires, Groupe Hospitalier Diaconesses Croix Saint-Simon, Paris, France.; 3 Unité de Recherche Clinique Paris Île-de-France Ouest, Hôpital Ambroise Paré (AP-HP), Boulogne-Billancourt, France.; 4 Laboratoire de Microbiologie, Hôpital Ambroise Paré (AP-HP), Boulogne-Billancourt, France.; 5 UMR 1173, UFR Simone Veil, Université de Versailles Saint-Quentin-en-Yvelines, Montigny-le-Bretonneux, France.; 6 Service de Microbiologie, Groupe Hospitalier Diaconesses Croix Saint-Simon, Paris, France.; 7 Laboratoire de Microbiologie, Hôpital Raymond Poincaré (AP-HP), Garches, France.

**Keywords:** prosthetic joint infection, follow-up, multiplex antibody detection, biomar

## Abstract

**Introduction:** Multiplex-antibody detection has been recently proposed for the noninvasive diagnosis of staphylococcal prosthetic joint infection (PJI). We evaluated this approach for the post-treatment follow-up of patients.

**Methods:** Nineteen cases of staphylococcal PJI were prospectively followed for one year after treatment. The IgG response against eight staphylococcal antigens was measured before surgery and one year post-surgery using Luminex technology (Austin, TX, USA); median fluorescence intensity values determined for each antigen were transformed into a “Total Response Index” (TRI).

**Results:** Patients (11 women/8 men) had a mean (SD) age of 72.2 (12.4) years. Site of prosthesis was the knee (n=10), the hip (n=8) and the shoulder (n=1). Ten patients were infected by *S. epidermidis*, six by *S. aureus*, and three by *S. lugdunensis*. TRI values at one year were significantly lower than pre-surgery values (mean [SD]: 5.9 [1.8] *versus* 8.1 [3.4], *p*=0.02) and decreased, on average, by 21.2%. TRI values markedly increased in two patients. One patient had a relapse of *S. aureus* PJI at five months post-surgery, with a 37% increase of the TRI. The other had septic failure three months after revision for *S. lugdunensis* PJI; all intraoperative samples remained culture-negative, but the TRI increased by 51% and the antibody profile showed a marked change, suggesting a reinfection with another staphylococcal species.

**Conclusion:** Multiplex-antibody measurement may be useful for the follow-up of staphylococcal PJI and may help to detect septic failure involving organisms targeted by the assay.

## Introduction

Prosthetic joint infection (PJI) is a major complication that affects 2 to 3% of arthroplasty placements and is a growing public health concern 10, 11. The management of PJI requires a multidisciplinary and standardized approach to obtain a reliable microbiological diagnosis and facilitate the choice of surgical strategies and appropriate antibiotic therapy.

Despite various well-adapted treatment options for PJI, therapeutic failure is a challenging problem 3. Revision surgery for PJI is associated with an up to four-fold increase in the risk of PJI 15. The risk of failure increases with the number of surgical recoveries and can rise to more than 50% for some patients 3. A number of risk factors have also been identified, including the involvement of a resistant organism, chronic lymphoedema of the affected extremity, and psychiatric disorders 15, 23.

In this context, there is a need to identify “predictors and early tools of failure” 7, 12. Follow-up of PJI patients consists of regular return visits for clinical and radiographic evaluation for one year postoperatively. The diagnosis of septic failure is usually based on clinical signs of infection, such as pain, joint swelling, fever, drainage, disunion of the surgical wound, or the presence of a draining sinus communicating with the joint. Septic failure may also be suspected based on the elevation of previously normal markers, such as the erythrocyte sedimentation rate (ESR) and/or C-reactive protein (CRP) 17. However, ESR and CRP are markers of inflammation and may re-increase in conditions other than infection. The variance of these markers has been reported to be too high to support clinical judgments in monitoring the progress of infection 1, 12. Serum procalcitonin and IL-6 have also been used as follow-up biomarkers, but with contradictory results 2, 6, 8 and no obvious benefit over ESR or CRP 20.

A new approach based on multiplex-antibody detection in serum has been recently proposed for the noninvasive diagnosis of PJI 14. This approach has been shown to have very good sensitivity for the three most frequent staphylococcal PJI pathogens: *S. aureus, S. epidermidis* and *S. lugdunensis* 14, 21. Moreover, it provides information distinct from that of ESR and CRP, as it can, for example, detect staphylococcal PJI when ESR and CRP values are normal 14. Here, we aimed to determine whether this new diagnostic approach may also be useful for the post-therapeutic follow-up of patients.

## Materials and methods

### Study population

We included all patients enrolled in a recent non-interventional prospective evaluation of a multiplex antibody detection-based immunoassay for the diagnosis of PJI 14 who: i) had surgery for PJI involving a single staphylococcal species consisting of *S. aureus*, *S. epidermidis*, or *S. lugdunensis*; ii) were followed for at least one year after surgery; and iii) had at least one serum sample taken at the inclusion visit before surgery and another at the one-year follow-up visit.

The protocol and information sheet were approved by the IRB “*CPP Île-de-France XI*”. The database was authorized by the “Commission Nationale Informatique Libertés” (French privacy watchdog) and all patients were informed before inclusion and given the possibility to opt out. The study was performed in accordance with the principles of the Declaration of Helsinki and Guidelines for Good Clinical Practice.

### Definitions

PJI was defined as previously described 14 by (i) the presence of a sinus tract and/or (ii) at least one intraoperative sample positive in culture with a virulent organism or at least two intraoperative samples positive in culture with the same microorganism (same species and same susceptibility profile). The absence of infection was defined as no sinus tract and no positive culture for any sample or a single culture positive for a nonvirulent organism. This definition takes into account the major infection criteria of the Infectious Diseases Society of America (IDSA) and the Musculoskeletal Infection Society (MSIS) guidelines 16, 18.

Septic failure was defined according to the Delphi criteria by the presence of at least one of the following criteria : presence of at least one clinical sign of PJI (evidence of fistula, healing wound or drainage); synovial fluid and/or intraoperative samples positive in culture; further surgical intervention for infection in the joint of interest; and subsequent PJI-related mortality 5. Diagnosis of aseptic failure was established when the patient had local pain, with no fistula nor healing wound and negative culture of synovial fluid and intraoperative samples.

A relapse was defined as a recurrence involving the same strain that was present before therapy. A reinfection was defined as a recurrence involving a different species or strain.

### Multiplex antibody detection

We used a bead-based multiplex assay (Magplex™ beads, Luminex, Austin, TX, USA) designed to target three staphylococci (*S. aureus, S. epidermidis, S. lugdunensis*). This assay measures serum IgG against a panel of eight purified recombinant staphylococcal antigens. Serum samples were diluted 1:70. IgG binding was detected with R-phycoerythrin-conjugated AffiniPure goat anti-human IgG (MOSS substrate, Pasadena, CA). Positive control, negative control, and calibrating sera were included in each series. Median fluorescence intensity (MFI) values were determined for each antigen using a Magpix instrument and Xponent software. MFI values were then transformed into a “Total Response Index” (TRI) using proprietary software (Diaxonhit, France) 14.

### Statistical analysis

Quantitative continuous variables were compared using Student's *t* test. *P* values of < 0.05 were considered statistically significant.

## Results

### Patient characteristics

A total of 19 patients were included, consisting of 11 females and eight males, with a mean age of 72.2 years (Table 1). Most patients (n = 13, 68.4%) underwent at least one previous implant revision and PJI occurred, on average, 2.9 years after the last implant insertion. Site of prosthesis was the knee (n=10), the hip (n=8) and the shoulder (n=1). Only one patient received an immunomodulating drug (tyrosine kinase inhibitor) for the treatment of chronic myeloid leukemia and two patients presented systemic inflammatory disease (leukodystrophy and gout). Ten patients were infected by *S. epidermidis*, six by *S. aureus*, and three by *S. lugdunensis*.

### Surgical and medical therapy

All patients were treated with one-stage exchange, except for three (case n°5, debridement and implant retention; case n°16, permanent resection arthroplasty; and case n°18, two-stage exchange arthroplasty).

All patients received parenteral antimicrobial therapy for a median duration of 15 days (range, 3 to 90 days). Vancomycin was the most frequently used antimicrobial agent (13/19, 68%).

All but three patients received oral antimicrobial therapy (median duration, 65 days; range, 38-90 days). Rifampicin was used in 79% of cases, fluoroquinolone in 63%, clindamycin in 21%, and minocycline in 16%. The most used combination was fluoroquinolone + rifampicin (nine cases, 56%).

### Follow-up

Six patients had early surgical recovery for aseptic (cases n°13, 16, and 17) or septic (cases n°1, 2, and 8) failure (Table 2). The three patients with septic failure had reinfection (Table 2). The six patients showed a good evolution at one year follow-up.

Two patients were re-operated for septic failure between 3 and 12 months post-surgery. The first patient was a 73-year-old woman with bacteremic *S. aureus* PJI of the right knee (case n°5, Table 2). She had open irrigation and debridement with retention of the implant and received iv. cloxacillin and ofloxacin followed by oral ofloxacin and rifampicin for five weeks. She was re-operated five months later for septic failure. The five intraoperative specimens yielded a rifampicin-resistant *S. aureus* strain otherwise indistinguishable from the original isolate. The patient then underwent knee arthrodesis with an armed spacer on two nails and was treated with daptomycin for seven days followed by five weeks of oral therapy with ofloxacin and clindamycin. The patient appeared free of active infection at one year follow-up.

The second patient was a 77-year-old woman with *S. lugdunensis* PJI of the right hip (case n°18, Table 2). She had two-stage exchange arthroplasty and received iv cefazolin and vancomycin for one month followed by oral levofloxacin and rifampicin for eight weeks. She was referred back to her surgeon three months later with pain and a discharging fistula. Hip aspiration yielded a purulent fluid but remained culture-negative. The patient underwent repeated debridement and was simultaneously benefited from reimplantation of her total right hip arthroplasty, with negative intraoperative microbiological findings. One year after reimplantation, the patient was asymptomatic and showed no sign of implant dysfunction.

### Kinetics of antibody response

**Figure 1:**
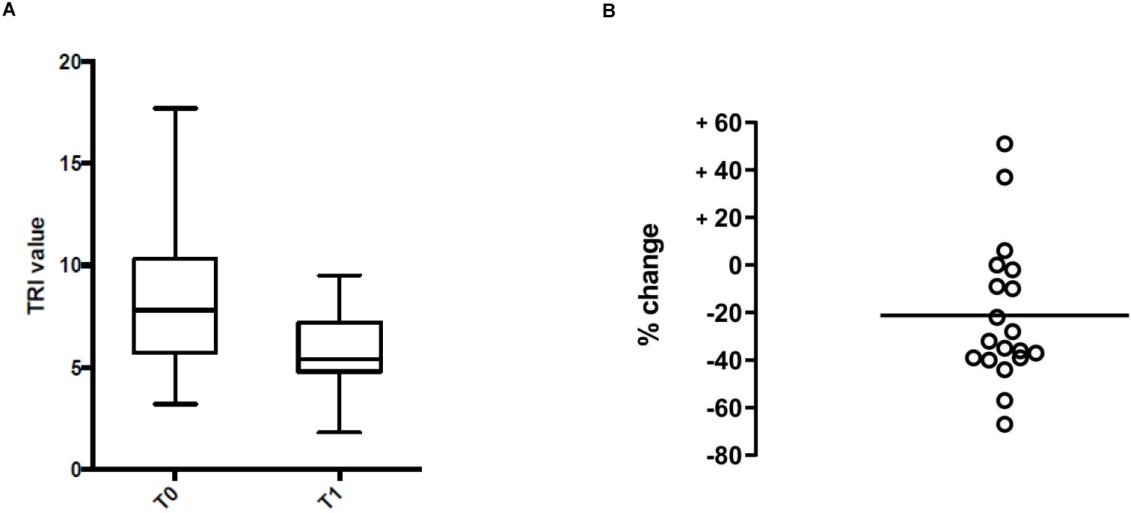
** Kinetics of the antibody response measured before surgery (T0) and one year post-surgery (T1). A.** Median and IQR values. Each rectangle spans the first quartile to the third quartile (the interquartile range = IQR). Bars indicate the median and minimum and maximum range. **B.** Changes in TRI. Circles represent the positive (increase) or negative (decrease) of TRI values (in percent) for each case. The bar indicates the mean change.

Overall, TRI values measured one year post-surgery were significantly lower than pre-surgery values (mean [SD]: 5.9 [1.8] *versus* 8.1 [3.4]; *p* = 0.02) (Figure 1A), with a mean (SD) decrease of 21.2 (29.9)% (Figure 1B). Only two patients showed a marked increase of their TRI values (case n°5: 37%; case n°18: 51%) (Figure 1B).

**Figure 2:**
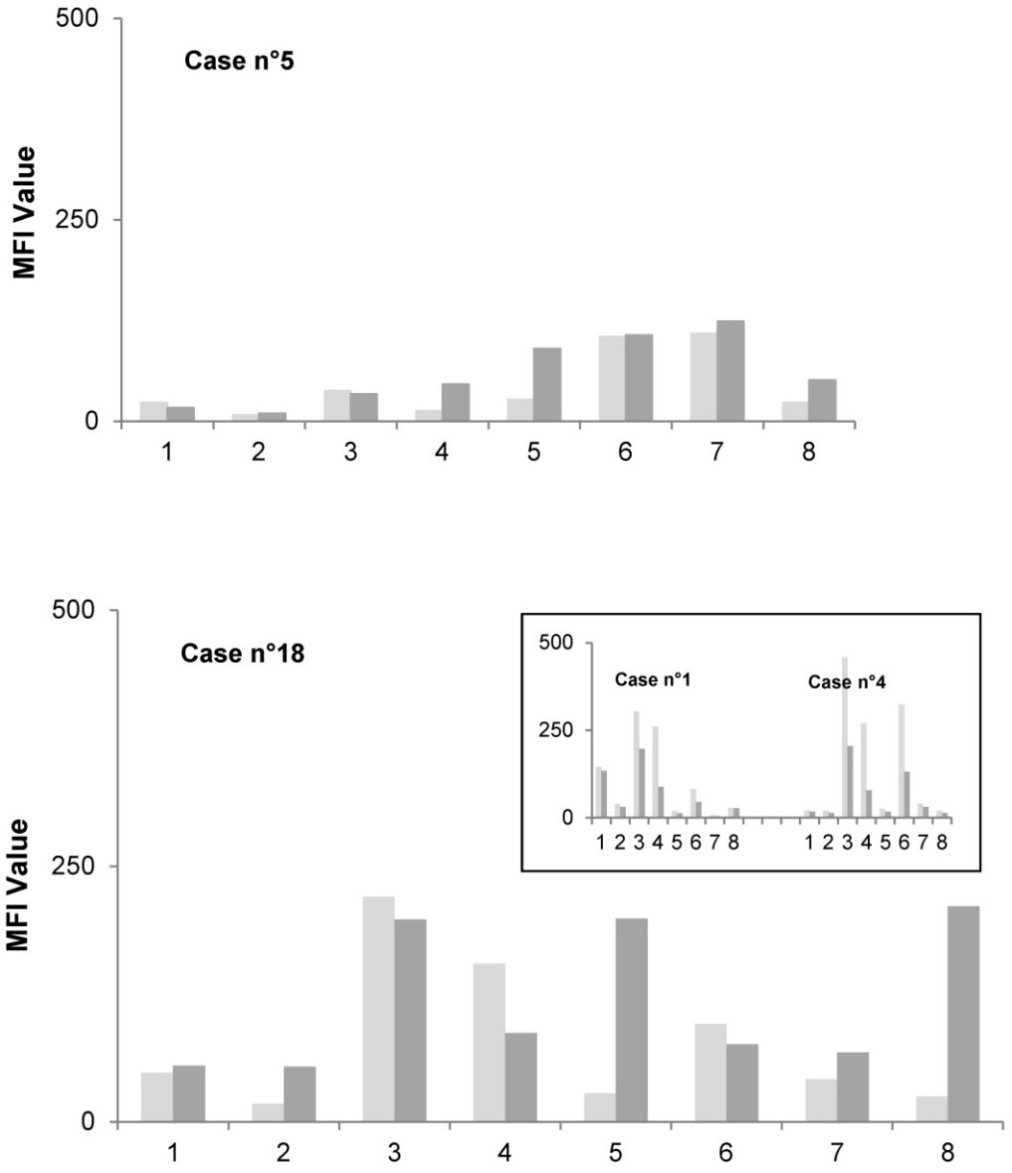
** Response profiles of cases n°5 (A) and n°18 (B).** The MFI values determined for each antigen (Ag) before surgery (T0, light gray) and one year post-surgery (T1, dark gray) are shown. Box: response profiles of *S. lugdunensis* cases °1 and n°4, note the lack of a response against antigens 5 and 8 in both cases.

We analyzed the kinetics of the antibody response for each of the eight antigens in cases n°5 and n°18. The antibody response at one year was directed against the same set of antigens as before surgery for case n°5 (see antigens 6 and 7, Figure 2A), consistent with the isolation of the same organism (*S. aureus*) at revision for septic failure five months after the first PJI episode (Table 2). In contrast, the antibody response profile showed a marked change in case n°18, for which the patient had septic failure with culture-negative intraoperative samples three months after *S. lugdunensis* PJI (Table 2). Indeed, the pre-surgery antibody profile showed a strong response against antigens 3, 4, and 6, whereas the post-surgery profile associated a decrease in antibody levels against these three antigens with the development of a strong response against two new antigens: antigens 5 and 8 (Figure 2B). Antigens 3, 4, and 6, but not 5 and 8, were recognized in the two *S. lugdunensis* PJI cases of our series with favorable outcome (Figure 2B, box), suggesting that the failure in case n°18 involved a staphylococcal organism other than *S. lugdunensis*, while the decreasing response to antigens 3, 4, and 6 was indicative of the successful treatment of *S. lugdunensis* infection.

Pre- and post-surgery CRP values were obtained at the same time as those for the TRI in 11 cases (Table 2). CRP and TRI kinetics were comparable overall, with a notable discrepancy for the two cases with an unfavorable outcome: decrease of CRP values to 6 mg/L and 14 mg/L at one year post-surgery for cases n°5 and n°18, respectively (Table 2). This discrepancy may be due to the shorter half-life of CRP than the antibodies measured (see below).

## Discussion

The results of our study show that multiplex-antibody measurement is an approach of potential interest for the post-therapeutic monitoring of staphylococcal PJI. Indeed, there was a very good correlation between the kinetics of anti-staphylococcal antibody levels and evolution after treatment (*i.e.*, a decrease in the level of antibodies in successful cases and an increase in cases of septic failure). In our series, the two cases of septic failure observed between 3 and 12 months post-surgery were both associated with a strong increase in the antibody response. In a single patient, a minor TRI increase of 6%, well within the inter-run variability of the method (<20%), was noted.

The results observed in our case of septic failure three months after the first of a two-stage treatment of a *S. lugdunensis* PJI suggest that the antibody response profile (*i.e.*, the number of antigens recognized and the intensity of the response to each) could provide valuable information about the causative organism. Indeed, the antibody response of this patient after the second episode was directed against antigens distinct from those initially recognized (strong reaction against two new antigens) and also distinct from those recognized in our two other cases of PJI with *S. lugdunensis*. This change in profile suggests a reinfection with a staphylococcus other than *S. lugdunensis*, without it being possible to specify the species in question (see the limits of the study below).

In spite of its interest, the "multiplex antibody measurement" approach nevertheless presents a number of limitations. First, this approach is dependent on the relevance and quality of the antigens used. We used a set of eight antigens already evaluated in the diagnosis of staphylococcal PJI that cover the three main species of staphylococci responsible for PJI (85 to 90% of all staphylococcal PJI) 22: *S. epidermidis*, *S. aureus*, and *S. lugdunensis*. Due to their cross-reactivity within the genus *Staphylococcus* 14, these same antigens could also be relevant for the post-therapeutic follow-up of PJI due to other less frequently occurring staphylococci, such as *S. capitis* or *S. caprae*, but this remains to be demonstrated (see the limits of the study below).

A second limitation of this approach is related to the relatively long half-life of antibodies in the blood. Indeed, the test relies on the measurement of IgG class antibodies, of which the half-life is generally estimated to be between three and four weeks 13. In comparison, the half-life of CRP is only 19 hours 19. The anti-staphylococcal antibody approach is therefore not superimposable with that of CRP for the post-therapeutic follow-up of patients and cannot substitute CRP. This is very well illustrated by the apparent discrepancies observed between antibody and CRP levels in the two cases of septic failure (high TRIs and normalized or almost normalized CRP levels one year post-surgery). However, the antibody approach is more specific than the measurement of CRP or VS and may indicate an infectious process if antibody levels again increase, either with the same profile as before or with a different profile, as for case n^o^ 18 of our series.

Our study also has other limitations and leaves a number of question open. The first concerns the slope of decrease of the antibodies, highlighted by the test in a real situation. In this pilot study, we only examined one follow-up sample one year post-surgery and observed the correlation of the antibody levels decrease with the clinical outcome. This study should be completed by a larger longitudinal prospective study in different centers to assess the operability of the test. It would be very useful to analyze samples taken at shorter intervals (1, 3, 6, 9 and 12 months postoperatively). The study of the kinetic of antibodies will provide the optimal sampling schedule to allow the prediction of success or failure in order to achieve optimal patient follow-up and help the clinicians take early and informed action the in real-time follow-up of their patients.

In the same way, it would be very informative to study the kinetics of antibody decay in cases of two-stage exchange arthroplasty. Several studies have attempted to identify markers to better define reimplantation timing but with disappointing results 4, 9. The antibody kinetics could provide insights into the opportunity of reimplantation if the rate of antibody decay is sufficiently rapid.

A higher number of cases would also help to address the question of the universal or non-universal nature of the test for staphylococcal PJIs, *i.e.,* the ability of the test to detect measurable antibodies regardless of the staphylococcal species. This would also provide a database to determine whether certain response patterns can indicate a particular species, particularly *S. aureus*. In addition, other complementary antigens could be included to obtain more discriminant profiles.

In conclusion, the measurement of the antibody response against relevant antigens appears to be a promising tool for the post-treatment follow-up of staphylococcal PJI. This approach should be studied in larger cohorts of patients to measure the early kinetics of anti-staphylococcus antibodies and to establish relevant threshold values. Its usefulness should also be evaluated as a decision support for second-stage reimplantation.

## Authors Contributions

T. Bauer participated to the design of the study, analyzed the results and participated to the writing of the manuscript.

S. Marmor participated to the design of the study, analyzed the results and participated to the writing of the manuscript.

I. Ghout performed the statistical analysis and participated to the writing of the manuscript.

E. Salomon performed the bacteriological study, analyzed the results and participated to the writing of the manuscript.

F. El Sayed performed the bacteriological study, analyzed the results and participated to the writing of the manuscript.

M Rottman designed the study, participated to acquisition of the data, analyzed the results and wrote the manuscript.

JL Gaillard designed the study, participated to acquisition of the data, analyzed the results and wrote the manuscript.

AL Roux designed the study, participated to acquisition of the data, analyzed the results and wrote the manuscript.

The study was accepted by an ethical review committee on the May 14^th^ 2012.

The work was performed in the two reference centers of bone and joint infections of Ile de France.

## Figures and Tables

**Table 1:** Characteristics of the studied population.

Characteristics (n=19)	
Mean (SD*) age, years	72.2 (12.4)
Female, no. /total no. (%)	11/19 (57.9)
Number of previous revision arthroplasties, no. /total no. (%)	
0	6/19 (31.6)
1	7/19 (36.8)
> 1	6/19 (31.6)
Site of prosthesis, no. /total no. (%)	
Hip	8/19 (42.1)
Knee	10/19 (52.6)
Shoulder	1/19 (5.3)
Mean (SD) time elapsed since prosthesis insertion, years	2.9 (4.9)
Diabetes mellitus	5/19 (26.3)
Immunosuppressive drug	1/19 (5.2)
Systemic inflammatory disease	2/19 (10.5)
Causative organism, no. /total no. (%)	
* Staphylococcus epidermidis*	10/19 (52.6)
* Staphylococcus aureus*	6/19 (31.6)
* Staphylococcus lugdunensis*	3/19 (15.8)

*SD : Standard deviation.

**Table 2:** Kinetics of antibody response and clinical follow-up.

Case n°	Organism	T0/T1^a,b^ TRI	T0/T1^a^ CRP^c^	Follow-up
1	*S. lugdunensis*	8.9/5.4 (-39%)	6/ND	Revision at 2 weeks for cicatricial necrosis (*Escherichia coli* from 1 intraoperative sample)
2	*S. epidermidis*	3.2/1.8 (-44%)	227/13	Surgical reoperation at 2 weeks for fistula (*Staphylococcus hominis* from 2 intraoperative samples)
3	*S. aureus*	6.7/4.8 (-28%)	139/<2	Nothing of note
4	*S. lugdunensis*	11.8/5.1 (-57%)	49/42	Nothing of note
5	*S. aureus*	3.5/4.8 (+37%)	11/6	Relapse of *S. aureus* PJI (rifampicin-resistant *S. aureus* mutant from 5 intraoperative samples)
6	*S. epidermidis*	8.2/8.7 (+6%)	11/1	Joint still painful at 6 months
7	*S. aureus*	7.2/4.7 (-35%)	105.4/ND	Nothing of note
8	*S. epidermidis*	5.7/3.9 (-32%)	1.1/ND	Revision at 2 weeks for disunion and effusion from the surgical wound (*Enterobacter aerogenes* from 5 intraoperative samples)
9	*S. aureus*	17.7/5.9 (-67%)	76.5/ND	Nothing of note
10	*S. epidermidis*	5.1/5 (-2%)	21/1.6	Nothing of note
11	*S. epidermidis*	11.3/5.5 (-40%)	8.6/<5	Nothing of note
12	*S. epidermidis*	8.6/7.7 (-10%)	25.6/19.8	Nothing of note
13	*S. epidermidis*	9.2/7.2 (-22%)	32.7/5.4	Revision at 2 weeks for disunion of the surgical wound (negative intraoperative samples)
14	*S. aureus*	6.6/4 (-39%)	39.8/ND	Nothing of note
15	*S. epidermidis*	5.3/3.9 (-9%)	1.9/ND	Nothing of note
16	*S. aureus*	7.8/7.8 (0%)	46.9/11.8	Revision at 4 weeks for disunion of the surgical wound (negative intraoperative samples)
17	*S. epidermidis*	10.9/6.9 (-37%)		Revision at 1 week for hematoma (negative intraoperative samples)
18	*S. lugdunensis*	6.3/9.5 (+51%)	27.3/14	Failure with sinus tract of the right hip. Revision prior to 2^nd^ stage
19	*S. epidermidis*	10.3/6.6 (-36%)	8.3/ND	Nothing of note

^a^ T0: pre-surgery value; T1: value at one year post-surgery. ^b^ (): % change. ^c^ In mg/L. ND, not determined.
